# Advancing health system strengthening through improving access to medicines: A review of local manufacturing policies in Ghana

**DOI:** 10.1177/2399202620962299

**Published:** 2020-10-12

**Authors:** Kwame Peprah Boaitey, Chloe Tuck

**Affiliations:** 1 Pharmaceutical Society of Ghana, Accra, Ghana; 2 School of Health and Related Research (ScHARR), University of Sheffield, Sheffield, UK; 3 Commonwealth Pharmacists’ Association (CPA), London, UK

**Keywords:** Local medicines manufacturing, medicines quality assurance, substandard and falsified medicines, medicines supply, health systems strengthening, Ghana

## Abstract

Providing access to quality-assured medicines is a fundamental component of strengthening health systems. Yet, the World Health Organization (WHO) estimates that 13.6% of all medicines in low- and middle-income countries (LMIC’s) may be substandard or falsified (SF) impeding patient outcomes, imposing financial burden, and contributing to antimicrobial resistance. Circulation of SF medicines also undermines trust in the health system and legitimate health care professionals. It may erode trust in the manufacturers of genuine pharmaceutical products as well as health professionals who prescribe and dispense them. Failure to address challenges in medicines quality assurance and supply risks jeopardizing progress towards universal healthcare coverage. This editorial draws on perspectives from a Ghanaian context and highlights the importance of ensuring an adequate and stable medicine supply, specifically through mechanisms to foster local manufacturing. This will serve to address the problem of SF medicines, as well as providing opportunities for mutual benefit with multiple related sectors. The WHO’s mechanism on substandard and falsified medical products 2020 highlights multiple sectors have a key role in combatting SF medicines. Although key considerations and initiatives in other sectors are beyond the scope of this article, local manufacturing should be viewed with WHO’s a multilevel systemwide approach.

## Medicines regulatory control as a tool to assuring the quality of medicines

Policies to ensure stringent regulation and governance of medicines supply overseen by a national medicines regulatory agency (NMRA) are imperative to ensuring their quality and safeguard patients from SF medicines.^1,2^

The importance of legal frameworks in enabling regulatory enforcement has been widely acknowledged,^2–7^ this underpinned the development of model laws, such as the African Medicines Regulatory Harmonization (AMRH) initiative.^2,5,8^ Nevertheless, model laws alone are not sufficient to ensure effective regulatory processes in practice; they must be backed by effective regulatory action, governance, accountability, and transparency across the medical product lifecycle, including appropriate public access to data on registered quality-assured medical products, inspection outcomes, product recall^9^ with adequate capacity to implement acceptable global standards in regulatory oversights. A country’s capacity to implement regulation of medicines requires a sufficient regulatory and health professional workforce with the appropriate level of expertise, oversight, and communications,^4–7^ underpinned by the efficient administrative system^7,10^ and access to robust high-quality testing facilities and screening technologies.^7,10,11^

Although progress in strengthening regulatory systems is being made globally, such as through short messaging service (SMS), medicines authentication technology such as serialization and traceability,^12^ screening technology advances (examples include in the field testing in China)^11^, there remain critical challenges, and many nations are limited in their capacity to effectively assure the quality of the supply of medicines through their NMRA.^2–6^

Strengthening any one aspect of medicines provision alone is unlikely to be effective at significantly increasing medicines quality assurance.^13^ Moreover, a well-equipped and functioning regulatory system in isolation is not enough without a reliable supply of quality-assured medicines. It is critical to consider the consequences of supply issues on access for patients.^11^ Supply shortages are the result of multiple factors and include environmental, the healthcare delivery service, and the pharmaceutical industry that manufactures the medicines. Thus coherent multi-level approaches encompassing multiple aspects of medicines regulation and supply are required including national anti-counterfeit drug laws and legislation, registration and licencing, inspection and quality control, training of health professional personnel, price control, technological interventions, national alert systems, and public education and awareness campaigns in collaboration with multiple stakeholders involved in the medicine supply chain.^7^

## Framing quality medicines supply within a health system context

Quality medicines supply should be considered within the broad context of the health system. For example, when medicines are not affordable through the formal regulated health system due to prohibitive costs or lack of national health insurance schemes, patients will be driven to seek these through other routes, where regulatory oversight may be compromised and there is a greater risk that the medicine will be substandard or falsified.^14^ This has been highlighted in Morocco where unaffordable medicines were implicated in driving medicine’s trafficking from Algeria and sold at markets, where the quality and validity were unknown.^15^

In addition, when healthcare institutions are not able to procure the quantities to meet their local demands for medicines, it may lead to stockouts. This may hinder the ability of the facilities to serve their patients. This can occur against a backdrop of national programmes to support equitable access, (e.g. national health insurance schemes) if there is a lack of availability through central or regional medicine stores. The outcomes can include one or several adaptive mechanisms:

- Institutions look to procure medicines from other suppliers such as through the open markets but may have to balance conflicting challenges if there is a higher price or limited capacity to assure quality 10
- Institutions may have to pay additional costs to obtain medicines, meaning they are either required to charge patients more to cover these costs, or these additional opportunity costs must be passed on to another domain of their healthcare facility (potentially restricting access or quality of care in another domain)
- Institutions cannot provide medicines, so patients must seek medicines elsewhere

The outcome is that in many cases patients will not be able to access or afford medicines or are forced to buy them through an unregulated route, where they are less likely to be quality assured.

Tackling challenges of quality assurance, medicines supply, accessibility, and affordability is part of a wider need to strengthen the pharmaceutical industry in a manner that is oriented to service healthcare systems to better meet local demand. Key within this will be increasing awareness, building regulatory capacity, and enhancing mechanisms of supply chain management, incorporating innovations in the supply of medicines, but also ensuring medicines are *produced* to meet these demands.

## Aligning the pharmaceutical manufacturing industry to serve local healthcare demands

The global trade of medicines was estimated at $1.2 trillion in 2018.^16^ The African pharmaceutical market is expected to reach $45 billion in 2020, yet only 30 percent of medicines are produced on the continent with the rest imported mostly from China and India.

Imported medicines remain more challenging to stringently control quality assurance, opening opportunities to ambiguity in quality through additional variables in production and supply. For example, there is an asymmetry between the regulation of imported and exported medicines enforced by high-income countries (HICs) with stronger regulatory capacity. Regulations for quality control on exported medicines are often insufficient or simply lacking, relying on goodwill.^10,11^ This poses a strain on central medicine stores in many low and middle-income countries (LMIC) to enforce regulations and adhere to WHO recommended procurement procedures.

In addition, a review of procurement processes found that medicines supply in LMICs is often dominated by a small number of suppliers and intermediaries resulting in high mark-up and increased cost of generic medicines compared to HICs.^17^ Conversely, the manufacturing of medicines locally can not only increase a countries autonomy in medicines supply but allow tighter control of quality assurance and transparency in production.

This also opens the opportunity for technology transfer, medical innovation, and research to meet local needs. Targeting the manufacturing of essential medicines locally increases supply to sufficiently meet the most critical local demands. Thus, patients can access medicines through the routes that offer the greatest quality assurance. Across Africa, the proportion of locally manufactured medicines varies from 20%–30%. However, a viable local pharmaceutical manufacturing industry in Africa will not only impact the African health system and its capacity to respond to the health needs of the people but also will contribute to the overall socio-economic development of the continent. These benefits become more critical in the wake of the Covid-19 pandemic and may contribute to the preparedness of the continent in dealing with future pandemics through improving access to medicines.

Despite the clear positive aspects of building a local manufacturing infrastructure, some obstacles can be challenging to overcome. These include regulation, supply chain, market size, human resource constraints, inadequate infrastructure, high operating costs, weak links between local and international suppliers, and the high cost of local commercial capital.

The Pharmaceutical Manufacturing Plan for Africa adopted by the Conference of African Ministers of Health in April 2007 and endorsed by the Heads of State and Government in Accra in July 2007^18^ identified regulatory harmonization as a critical pillar for local manufacturing capacity in Africa. Since then, the African Union Commission has been at the forefront in galvanizing the necessary political will and providing the leadership to the broad range of processes required for promoting a sustainable local pharmaceutical industry. Progress is being made to harmonize regulation internationally and across Africa to maintain cross-country quality assurance, such as through the establishment of an African Medicines Regulatory Authority.^10^ The need to ‘Expand and maintain the global focal point network among national medicines regulatory authorities to facilitate cooperation and collaboration’ was stated in WHO’s 2020–2021 SF medicines priorities as part of their mechanism to prevent, detect, and response to ensure supply chain safety, although the role of local manufacturing was not recognized explicitly.^19^

Several countries such as Ethiopia, South Africa, Kenya, and Ghana have made considerable advances to boost the local pharmaceutical manufacturing sector.^20^ Very useful learnings can be derived from policies adopted by Ghana in comparison to those implemented elsewhere. These present potential comparative approaches by analysing the relative strengths, and weaknesses, in order to foster local pharmaceutical manufacturing as a mechanism to strengthen health systems through medicines access, supply, and quality assurance.

## Ghana as a manufacturing hub

Ghana is the second-most populous country in West Africa, with a population of 27.41 million. This is expected to increase to 36.87 million by 2030. Ghana is a LMIC and aspires to advance to a middle-income economy with a gross domestic product (GDP) per capita of US$3000 by 2020.^21^ Ghana’s industrial sector recorded a 10.6 percent growth rate in 2018 according to the Ghana statistical services report in April 2019.^22^

Ghana has made considerable healthcare reforms, establishing a National Health Insurance Scheme (NHIS) in 2004, which covers several essential medicines for acute and some chronic conditions.^23^ However, challenges remain at the health-care facility level in supplying medicines due to delays in repayments from the National Health Insurance Authority (NHIA) resulting in drug shortages, out-of pocket-payments,^24^ and patients risking catastrophic costs.^25^

The pharmaceutical sector is the key component of the healthcare system to meet the varying demand for pharmaceuticals. The total market size of the pharmaceutical market in Ghana is estimated at $329 million.^26^ This is made up of approximately 30% locally manufactured pharmaceuticals with the rest imported mainly from India and China for both prescription and over-the-counter medicines. This is despite the Ghanaian pharmaceutical industry being the second largest in the West African region with 38 registered pharmaceutical manufacturers.

In response to the call of African Heads of State for local production of essential medicines in Africa and the subsequent adoption of the Business Plan for accelerated implementation of the Pharmaceutical Manufacturing Plan for Africa (PMPA BP) in 2012, Ghana has made efforts to improve its pharmaceutical manufacturing capacity to meet local needs and that of the West African sub-region through the development of several policy frameworks. These include the Coordinated Programme of Economic and Social Development Policies (2017–2024),^27^ and the Medium-Term National Development Policy Framework (2018–2021).^28^ The Ghana Industrial Policy, National Health Policy, National Medicines Policy, Health Commodity Supply Chain Master Plan, and Industrial Sector Support Programme all aim at supporting the growth of the pharmaceutical sector.

With the support of the United Nations Industrial Development Organization (UNIDO), the National Development Planning Commission, the Pharmaceutical Society of Ghana, and various stakeholders, Ghana has developed the Ghana Pharmaceutical Sector Development Strategy (GPSDS) with the vision of making Ghana a world-class pharmaceutical investment and manufacturing hub, producing and supplying high-quality and affordable medicines for the people of the whole continent. A coordinated approach to these initiatives, backed by a strong political will has the potential of achieving this objective in the medium- to long-term.

These policy initiatives have also been backed by several efforts by the government including

- Restriction of some 49 products with local manufacturing capacity from importation (EI 181, May 2017);
- Expansion of the list of VAT-exempted pharmaceutical raw materials and inputs used for local pharmaceutical manufacturing to about 400 (LI 2218 of 2015);
- Removal of VAT/import tariffs on equipment for plant upgrades;
- Access to funding through the Export-Import (EXIM) bank.

Implementation of other initiatives including a 15% price preference for locally manufactured products for Government of Ghana procurement,^29^ the planned establishment of an industrial pharmaceutical manufacturing park with the support of the Ghana Chamber of Pharmacy and the Pharmaceutical Society of Ghana offers the platform to drive local manufacturing. Other pivotal initiatives include the formation of the Pharmaceutical Manufacturers Association of Ghana (PMAG)^30^ to serve as a platform for advocacy and to also provide a self-auditing system, a robust national Food and Drug Authority (FDA), and a stable democratic climate are key indicators which may ensure the achievement of the goal of becoming a pharmaceutical manufacturing hub.

These policies are anticipated to improve medicines supply through supporting local manufacturing of medicines, which will in tandem support the acquisition and use of medicines of higher quality assurance.

However, the impact these policy initiatives have on the supply chain has not yet been formally assessed. This remains an area where greater policy impact research is required to ascertain the most effective approaches going forward. Further light on this topic will highlight the intersectionality between economic development, medicines supply, affordability, and quality assurance at the point of care, which will support the policymaker looking for concrete evidence. This, in turn, will strengthen quality assurance and enable health systems to progress towards universal health coverage (UHC) in line with Sustainable Development Goal (SDG) 3 (*Good Health and Well-being*), and also build a prosperous economy to support peoples’ well-being as stipulated by SDG 8 (*Decent Work and Economic Growth*) and 9 (*Industry, Innovation, and Infrastructure*).^31^
